# Micro-Morphological Features of the Er:YAG-Lased Interface in Primary Teeth: 12 Months Randomized Split-Mouth Trial †

**DOI:** 10.3390/jfb15010017

**Published:** 2024-01-01

**Authors:** Osama Felemban, Raghdah Abdrabuh, Omar El Meligy, Najat Farsi, Ahmed Samir Bakry, Tariq Abu Haimed

**Affiliations:** 1 Paediatric Dentistry Department, Faculty of Dentistry, King Abdulaziz University, Jeddah 21589, Saudi Arabia; reabdraboh@kau.edu.sa (R.A.); omeligy@kau.edu.sa (O.E.M.); nfarsi@kau.edu.sa (N.F.); 2 Paediatric Dentistry and Dental Public Health Department, Faculty of Dentistry, Alexandria University, Alexandria 21521, Egypt; 3 Restorative Dentistry Department, Faculty of Dentistry, King Abdulaziz University, Jeddah 21589, Saudi Arabia; hbakry@kau.edu.sa (A.S.B.); tabuhaimed@kau.edu.sa (T.A.H.); 4 Department of Conservative Dentistry, Faculty of Dentistry, Alexandria University, Alexandria 21521, Egypt; 5 King Fahd Medical Research Center, King Abdulaziz University, Jeddah 21589, Saudi Arabia

**Keywords:** Er:YAG laser, bond interface, primary teeth, split-mouth

## Abstract

Despite considerable improvements in oral health, dental caries remains a public health issue. The most frequently used technique to remove caries is through rotating drills. New minimally invasive strategies were introduced into dental practice, such as the use of lasers to perform highly controlled tissue ablation while limiting pain and discomfort, as well as overcoming drill phobia. The objective was to assess and compare treatment with Er:YAG laser versus a conventional rotary treatment during cavity preparation in children with regard to bond interface quality. In a randomized trial using a split-mouth design, 40 (9–12 year-old) children with 80 carious primary molars were included. The cavity in one quadrant was treated conventionally using a bur, while the cavity in the other quadrant was prepared using an Er:YAG laser. Twenty restored teeth were extracted after one year. The SEM histological evaluation of bond interface results demonstrated no statistically significant differences between restorations placed following bur preparation and those placed following the Er:YAG laser preparation, and both treatments demonstrated promising results. Over a one-year period, no statistically significant differences in the bond interface quality were observed following class I cavity preparation in primary teeth with either Er:YAG laser or a conventional rotary bur.

## 1. Introduction

Laser devices have recently emerged as a viable alternative to classic mechanical rotating tools for the eradication of dental caries and cavity preparation [1]. There is a wide range of lasers that dentists utilize. The erbium-doped yttrium aluminum garnet (Er:YAG) laser (wavelength of 2940 nm) has been recognized as one of the approved alternative techniques for dental cavity preparation by the Food and Drug Administration (FDA) [2]. The absence of vibration, less pain, decreased danger of cross-contamination, selective caries eradication, and, in many situations, the elimination of LA administration are all advantages of using the Er:YAG lasers over the conventional rotating burs for dental treatments [3]. When used on dental hard tissues, laser energy is absorbed by the intrinsic H_2_O molecules and hydroxy groups (−OH) present in the apatite mineral. This absorption leads to the vaporization of the water content, resulting in the expansion and subsequent buildup of pressure within the dental hard tissues. Ultimately, this pressure buildup induces micro-explosions within these structures, leading to the removal of a small fraction of the dental hard tissue [4].

Previous research has indicated that the Er:YAG laser has promise for application in cavity preparation within the field of pediatric dentistry. This is attributed to the enhanced cooperation observed among young patients when undergoing dental cavity preparation using the Er:YAG laser [5,6,7]. The aforementioned benefit can be ascribed to the absence of vibration, noise, and forceful water jet commonly associated with traditional cavity preparation techniques. One of the additional benefits associated with the utilization of the Er:YAG laser is its bactericidal effect [8]. Nevertheless, it is important to take into account certain specific considerations when employing the Er:YAG laser for cavity preparation. This is because the ablation of dentin using the Er:YAG laser leads to the opening of dentinal tubules without causing any widening. Additionally, the intertubular dentin is ablated to a greater degree compared to the peritubular dentin, primarily due to its higher water and hydroxyapatite content. Consequently, the resulting dentin surface lacks any smear layer or smear plugs [9].

A previous study has indicated that the interface between the Er:YAG-lased dentin and adhesive may be susceptible to negative consequences resulting from pulpal pressure at the base of the prepared cavities [10]. This could potentially compromise the integrity of the adhesive restoration. Additionally, there is evidence suggesting the occurrence of fluid movement across the resin/dentin interface during and after the bonding process, which may lead to the occurrence of the nanoleakage [10,11,12]. The term nanoleakage, initially proposed by Sano et al., refers to microporosities with a size of less than 50 nm situated between the unmodified dentin and the resin-infiltrated collagen-rich fibrous network known as the “Hybrid Layer” [11]. Multiple studies have demonstrated that nanoleakage has the potential to compromise the integrity between the adhesive restorations and the walls of the dental-prepared cavities, ultimately resulting in the failure of the adhesive restoration [11,12,13].

The existing body of the literature offers a limited understanding of the long-term resilience of dentin treated with the Er:YAG laser and bonded with universal adhesives across many clinical scenarios [14]. The majority of prior investigations pertaining to the bond between resin and dentin in teeth that underwent various forms of cavity preparation and different types of restoration were conducted in laboratory settings [15]. In contrast, the current study focused on evaluating the bond interface quality of in vivo restorations of deciduous teeth. The aim of this study was to assess and compare the bond interface quality of cavities prepared using the Er:YAG laser (2940 nm) versus a conventional rotary treatment during cavity preparation in primary teeth. This study’s null hypothesis postulated that the resin/tooth contact would experience negative consequences as a result of pulpal pressure during the experimental clinical observation period. 

## 2. Materials and Methods

The Research Ethics Committee of the Faculty of Dentistry at King Abdulaziz University (KAU) approved this study’s proposal with the number 063–02–19. The patient population in the pediatric dental clinics at the King Abdulaziz University Dental Hospital (KAUDH) in Jeddah, Kingdom of Saudi Arabia, served as the source of study participants. Patients between the ages of 9 and 12 who were healthy and had at least two bilateral primary molars with simple occlusal caries limited to enamel and dentin but no proximal caries were included in the sample. The primary molars needed to have a third of their roots non-resorbed with permanent successors, with about half of their roots formed. If the primary molars had caries lesions that had reached or were close to the pulp, previous fillings, periapical lesions, dental abscesses, or if the subject had behavioral or medical issues, they were disqualified from this study.

An experiment using a split-mouth design that was randomized was carried out. Randomly, the children were allocated into one of two groups. The randomization was carried out using a sealed envelope technique to determine which method for cavity preparation was to be carried out first. Each participant was instructed to choose one of 40 identical sealed envelopes. The envelope was then opened, and the assignment sequence was read and followed before being discarded. Both groups underwent conventional caries removal and laser caries removal. The first group was given conventional caries removal on the first visit and laser caries removal on the second. A laser caries removal was performed in the second group at the first visit and a conventional caries removal was performed in the second group. All clinical operations were carried out by one of the authors (R.A.), who had received training in the biophysics of lasers as well as the methods and safety standards for utilizing them in dentistry. The conventional caries removal and the laser caries removal were administered over the course of two different appointments, spaced roughly one week apart. The restored teeth were extracted in accordance with the regular exfoliation schedule after a one-year follow-up period, and the bond interface quality was assessed then.

### 2.1. Treatment Procedures

**Figure 1:**
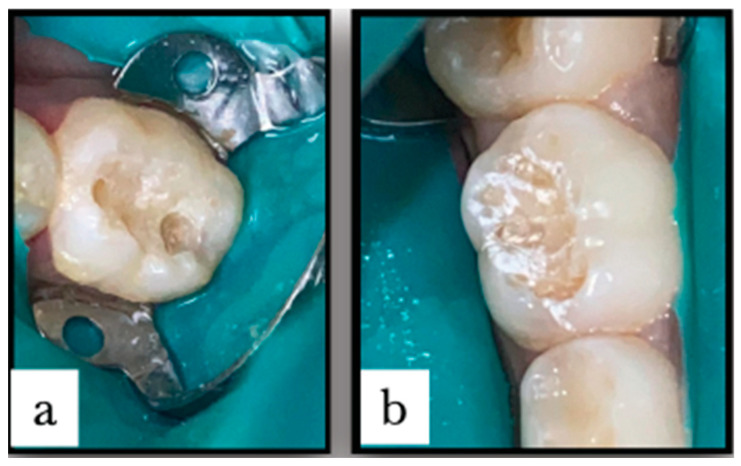
Caries removed and cavity prepared using (**a**) conventional method showing definite cavity outline and (**b**) Er:YAG laser method showing no definite configuration for the cavity outline due to laser ablation (Reprinted from Ref. [5]).

**Figure 2:**
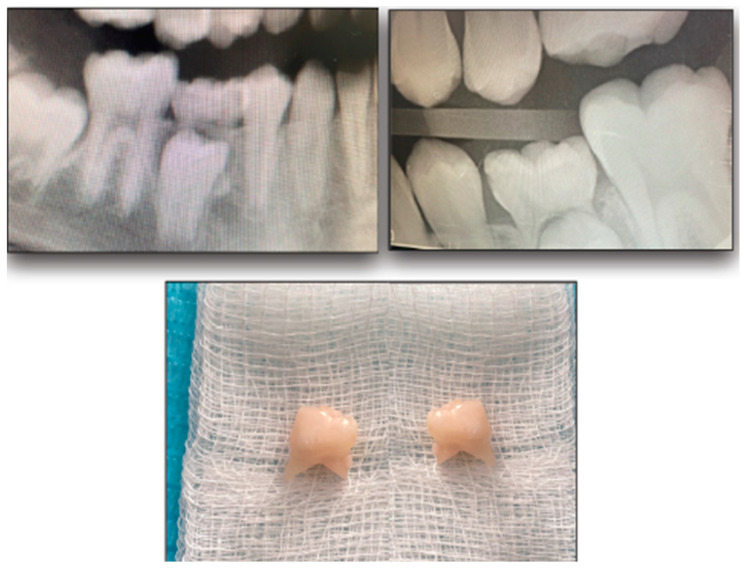
The restored teeth were extracted after determination of root resorption of the primary molar and adequate formation of the root of the successor tooth.

All teeth underwent complete isolation using a rubber dam and saliva ejector. Patients received topical anesthesia before the clamp was placed. In the conventional caries removal group, dental caries was excavated using the master torque high/low-speed air rotor handpiece (KaVo Dental, Charlotte, NC, USA) until visual inspection showed that the carious lesions had been thoroughly removed. In the laser caries removal group, dental caries was excavated using the Er:YAG laser with a wavelength of 2940 nm (Doctor Smile, Pluser, Brendola, Italy) according to the manufacturer’s instructions until visual inspection showed that the carious lesions had been thoroughly removed (Figure 1). In both treatments, if the subject experienced pain and requested local anesthesia, the procedure was stopped, local anesthesia was administered, and then the procedure was resumed. Cavities were restored using the Clearfil Universal Bond Quick system (Kuraray, Noritake Dental Inc, Tokyo, Japan) in total for each mode; 37% phosphoric acid was used; then, the Clearfil Universal Bond system was applied, light-cured (Woodpecker LED-F, Guilin Woodpecker Medical Instrument, Guilin, China) for 10 s, after which composite resin was applied (3M^TM^ Filtek^TM^ Z350 XT Universal Restorative, 3M ESPE) and light-cured according to the manufacturer’s instructions. One year later, the restored teeth were evaluated clinically and radiographically and were extracted after it was confirmed that the root of the included primary molars had resorbed and the roots of the successor tooth had formed (according to the patient’s typical tooth shedding pattern as shown in the example in Figure 2). 

### 2.2. Bond Interface Evaluation

**Figure 3:**
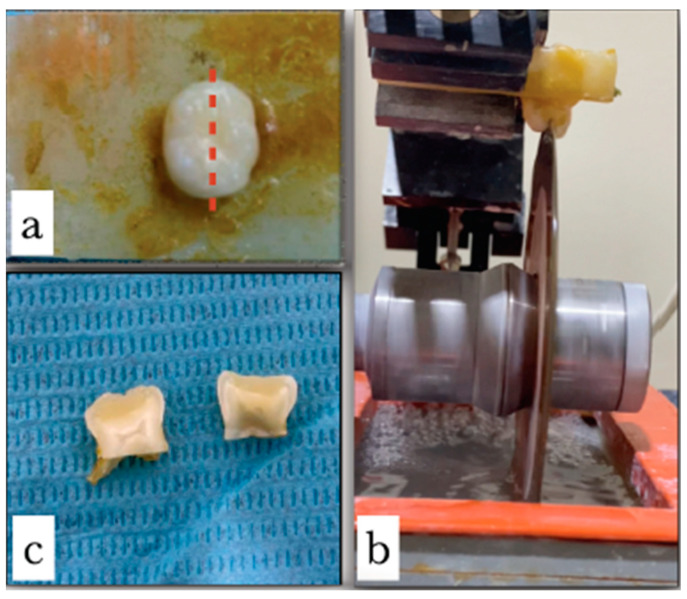
(**a**) The extracted tooth was prepared for sectioning along the mesiodistal direction (red dashed line). (**b**) Under-water lubrication; the extracted tooth has been vertically sectioned using a diamond saw. (**c**) The two central slabs are 3 mm thick.

The extracted teeth were first cleansed with water and disinfected with an antiseptic solution to eliminate any debris, germs, or blood. To preserve moisture and avoid desiccation, the teeth were stored in normal saline in a tightly sealed plastic container. The extracted teeth were then cut into 3 mm thick slabs using a diamond saw (1600 Microtome, Leitz; Wetzlar, Germany) while being irrigated with water. Two center slabs were produced and investigated further from each specimen (Figure 3). Wet #1000 Silicon Carbide Paper was used to grind and polish each slab. The process described by Tay, Pashley, and Yoshiyama (2007) was followed in order to prepare ammoniacal silver nitrate. For 18 h in complete darkness, each specimen was submerged in AgH_6_N_3_O_3_ [16]. The specimens were taken out of the AgH_6_N_3_O_3_, rinsed, and then placed in a photo-developing solution for 6 h under a fluorescent lamp. After being taken out of the photo-developing solution, the specimens were polished. The specimens were then subjected to a 5 min sonication procedure to eliminate the surface silver. With the use of the sputter coating method, the samples were given a gold coating to prepare for SEM imaging. In order to offer higher resolution, secondary electron images were used to evaluate the materials under a field emission SEM. The investigator who examined the SEM photos was unaware of which caries removal technique was depicted on which image.

### 2.3. Evaluation of Adhesive Interface Quality

A uniform distance was used to take photos of each segment for slides. The adhesive contact quality of slides was assessed for each of the following areas:

1. Integration of the enamel walls with the adhesive;
2. Integration of the dentin walls with the adhesive;
3. Integration of the dentin floor with the adhesive.

Each component was given a score between 0 and 1. One indicates a non-integrated adhesive interface, while zero indicates an integrated adhesive interface. Two experienced and calibrated examiners (academic professors from the operative dentistry department) assessed the specimens. Any disagreements in the evaluation were discussed until a consensus was achieved.

### 2.4. Statistical Analysis

For demographic categorical data, frequencies and percentages were presented, while mean and standard deviation were calculated for continuous data. The teeth were studied as pairs in order to account for the split-mouth design within the subject effect. The following categories were used to group the subjects: In the pair of teeth, the bond interface is successful in both teeth, unsuccessful in both, successful in the laser-treated tooth but unsuccessful in the conventional, or unsuccessful in the laser-treated tooth but successful in the conventional. At a significance level of 0.05, the McNemar test was utilized to analyze statistical differences between the laser and conventional methods. Statistical Package for Social Sciences software (IBM Corp. Released 2015. IBM SPSS Statistics for Windows, Version 23.0. Armonk, NY, USA) was used to analyze the data.

## 3. Results

**Figure 4:**
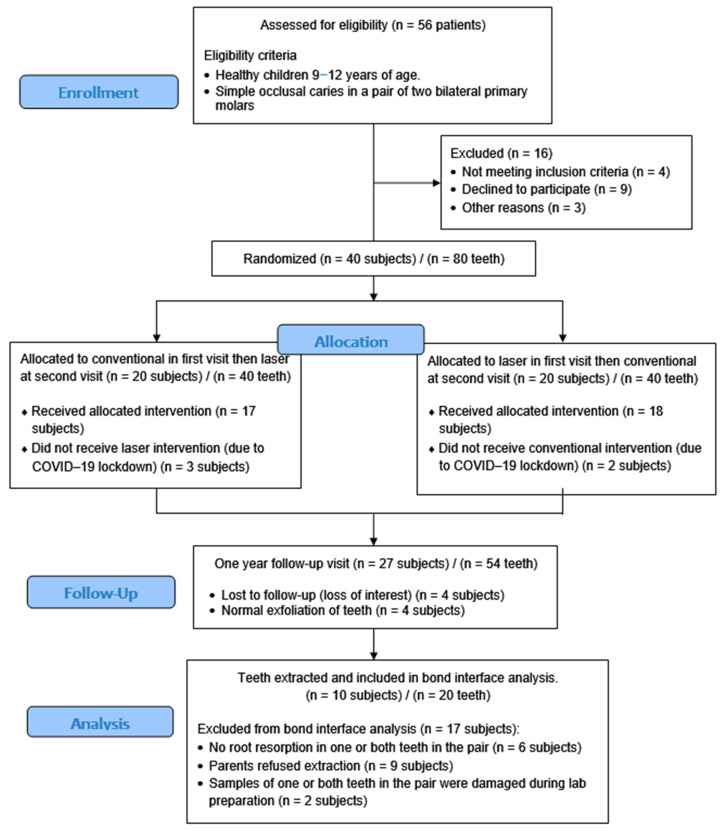
Flowchart of this study’s procedures.

A total of 56 participants were evaluated to determine their eligibility; 16 children patients were excluded from this study, and 40 subjects’ teeth were randomly assigned into one of the two groups after meeting the inclusion requirements. Five subjects, three from the first group and two from the second group, commenced this study but were unable to participate in the second visit of this study due to the COVID-19 lockdown and were subsequently excluded. At the one-year follow-up, four participants indicated they were no longer interested in continuing this study. Four additional subjects attended the one-year visit but had already experienced the loss of one or both of their included molars due to the natural exfoliation. A total of 27 subjects were available during the one-year follow-up visit. Out of the 27 subjects, 6 did not exhibit sufficient root resorption to warrant tooth extraction, while the parents of 9 subjects opted against extracting the molars. The molars of 12 participants were extracted for laboratory evaluation of the bond interface. However, the samples of two subjects were damaged during the preparation process. The final analysis of bond interface quality included the teeth of 10 subjects, totaling 20 teeth (Figure 4). The average age of the 10 participants was 9.5 ± 1.2, and 8 of them were males and 2 were females.

**Figure 5:**
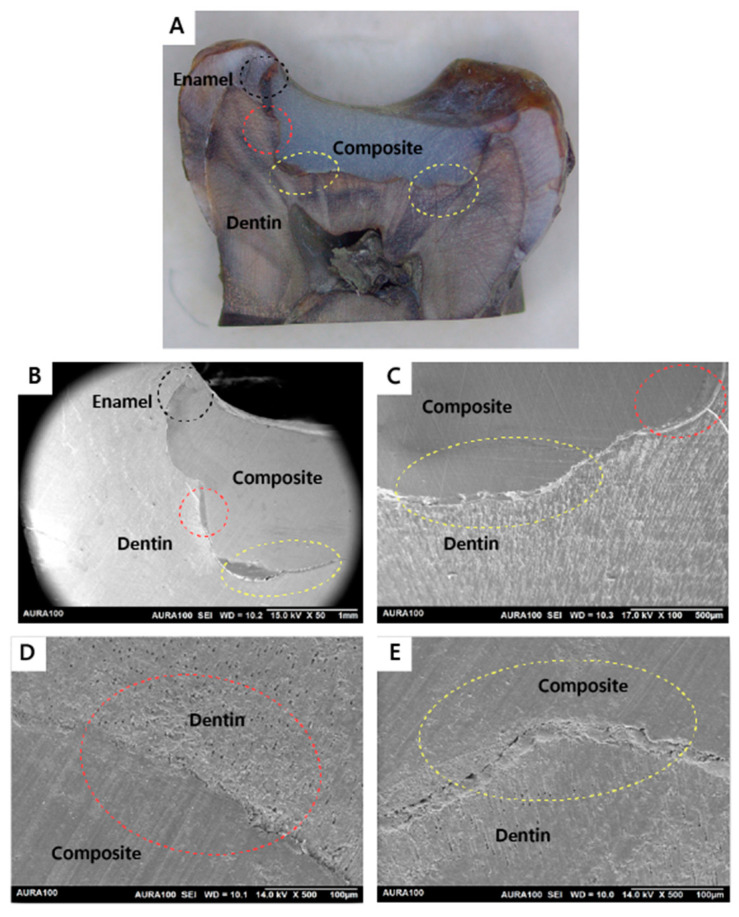
Sample prepared by the conventional method (integrated): (**A**) A stereomicroscope image showing the whole cavity and the restoration ablated by the conventional method. Cavity wall of enamel–composite interface (black circle). Cavity wall of the dentin–composite interface (red circle). Cavity floor of the dentin–composite interface (yellow circle); (**B**) SEM image showing the wall and the floor of restoration. Cavity wall of enamel–composite interface (black circle). Cavity wall of the dentin–composite interface (red circle). Cavity floor of the dentin–composite interface (yellow circle). All interfaces are integrated (magnified view ×50); (**C**) SEM image showing the cavity wall of the dentin–composite interface (red circle) and cavity floor of the dentin–composite interface (yellow circle). All interfaces are integrated (magnified view ×100); (**D**) SEM image showing the cavity wall of the dentin–composite interface (red circle) (magnified view ×500); (**E**) SEM image shows the cavity floor of the dentin–composite interface (yellow circle) (magnified view ×500).

**Figure 6:**
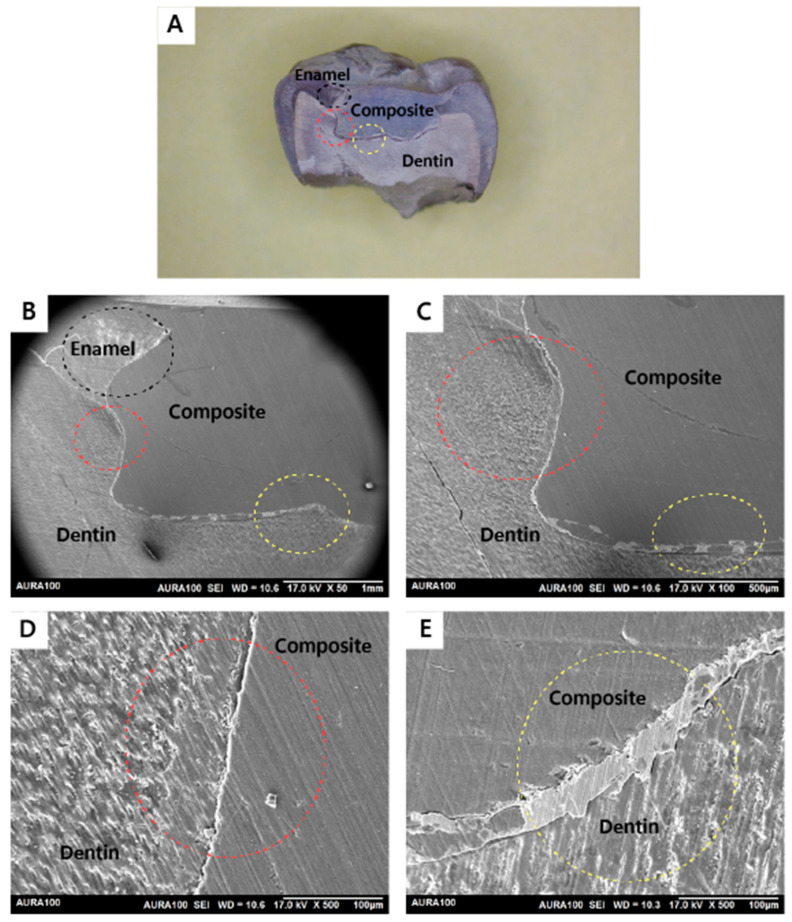
Sample prepared by the Er:YAG laser method (integrated): (**A**) A stereomicroscope image showing the whole cavity and the restoration ablated with the Er:YAG laser. Cavity wall of enamel–composite interface (black circle). Cavity wall of the dentin–composite interface (red circle). Cavity floor of the dentin–composite interface (yellow circle); (**B**) SEM image showing the wall and the floor of restoration. Cavity wall of enamel–composite interface (black circle). Cavity wall of the dentin–composite interface (red circle). Cavity floor of the dentin–composite interface (yellow circle). All interfaces are integrated (magnified view ×50); (**C**) SEM image showing the cavity wall of the dentin–composite interface (red circle) and the cavity floor of the dentin–composite interface (yellow circle). All interfaces are integrated (magnified view ×100); (**D**) SEM image showing the cavity wall of the cavity dentin–composite interface (red circle) (magnified view ×500); (**E**) SEM image shows the cavity floor of the dentin–composite interface (yellow circle) (magnified view ×500).

**Figure 7:**
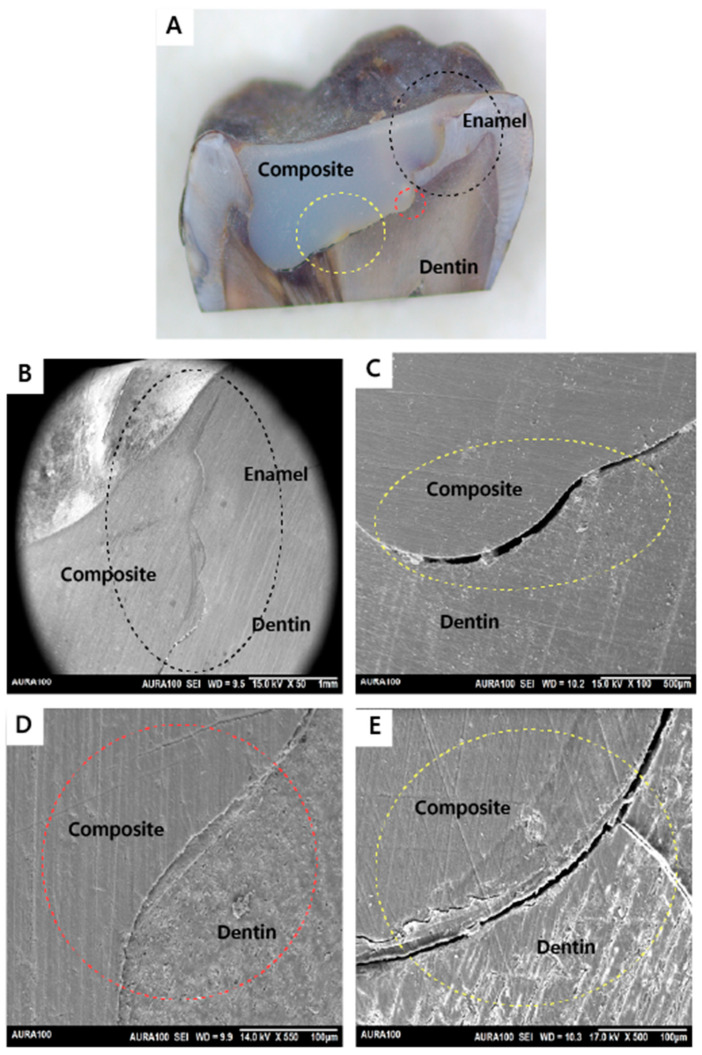
Sample prepared by the conventional method (not integrated): (**A**) A stereomicroscope image showing the whole cavity and the restoration ablated with the conventional method. Cavity wall of enamel–composite interface (black circle). Cavity wall of the dentin–composite interface (red circle). Cavity floor of the dentin–composite interface (yellow circle); (**B**) SEM image showing the cavity wall of the enamel–composite interface (black circle). The cavity wall of the enamel–composite interface is integrated (magnified view ×50); (**C**) SEM image showing the cavity floor of the dentin–composite interface (yellow circle). The cavity floor of the dentin–composite interface is not integrated (magnified view ×100); (**D**) SEM image showing the cavity wall of the dentin–composite interface (red circle). The cavity wall of the dentin–composite interface is integrated (magnified view ×500); (**E**) SEM image showing the cavity floor of the dentin–composite interface (yellow circle) (magnified view ×500).

**Figure 8:**
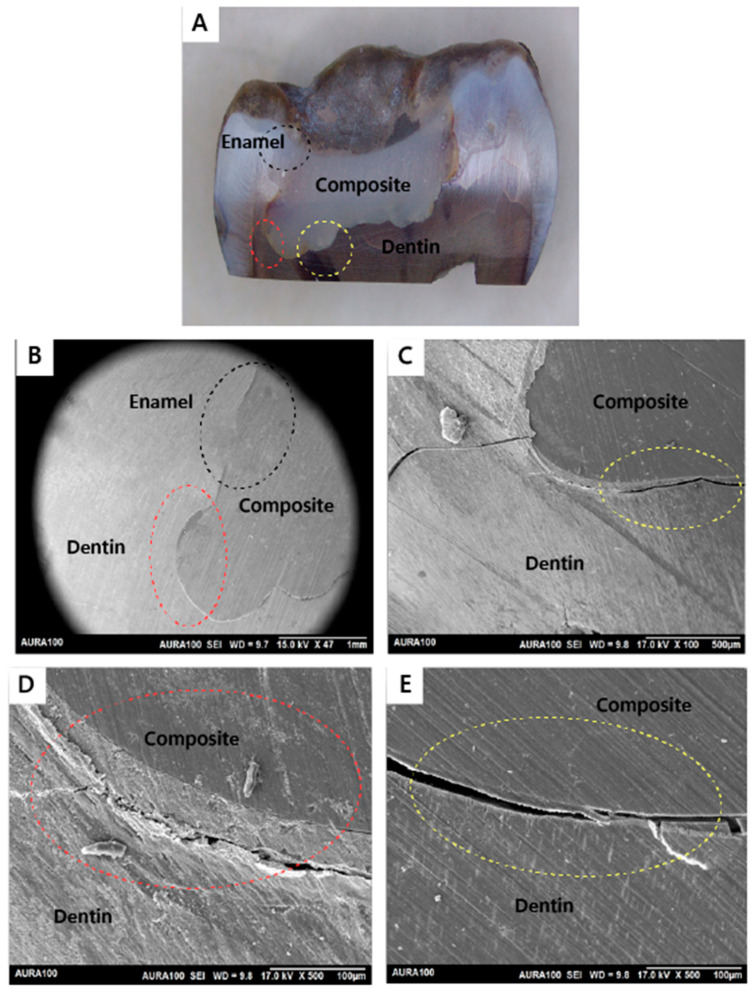
Sample prepared by the Er:YAG laser method (not integrated): (**A**) A stereomicroscope image showing the whole cavity and the restoration ablated by the Er:YAG method. Cavity wall of enamel–composite interface (black circle). Cavity wall of the dentin–composite interface (red circle). Cavity floor of the dentin–composite interface (yellow circle); (**B**) SEM image showing the cavity wall of the enamel–composite interface (black circle) and the cavity wall of dentin–composite interface (red circle). All cavity wall interfaces are integrated (magnified view ×50); (**C**) SEM image showing the cavity floor of the dentin–composite interface (yellow circle). The cavity floor of the dentin–composite interface is not integrated (magnified view ×100); (**D**) SEM image showing the cavity wall of the dentin–composite interface (red circle) (magnified view ×500); (**E**) SEM image showing the cavity floor of the dentin–composite interface (yellow circle) (magnified view ×500).

Out of 20 restorations, as assessed by SEM, all restorations (n = 20) were integrated at the enamel and dentin walls with the adhesive utilized in the restorations placed after caries removal with the conventional or the Er:YAG laser method. Regarding the adhesive integration with the dentin floor, six subjects had both of their restorations completely integrated. In contrast, only two subjects had a restoration that was not integrated on the dentin floor with the adhesive utilized in the restorations in the tooth prepared by the conventional method but the other restoration (prepared with laser) was integrated on the dentin floor. Also, only two subjects had a restoration that was not integrated on the dentin floor with the adhesive utilized for the restorations in the tooth prepared by the laser method, but the tooth that was prepared with conventional bur had a fully integrated bond on the dentin floor. The results showed no statistically significant difference in bond interface quality of the restorations placed after caries removal with the conventional or the Er:YAG laser method, with a *p*-value of 0.617 (Table 1). Figure 5, Figure 6, Figure 7 and Figure 8 demonstrate examples of the bond adhesive integration to enamel and dentin. 

## 4. Discussion

The present investigation is a randomized, controlled clinical trial, which permits within-subject comparisons due to its utilization of a split-mouth design, wherein each participant serves as their own control. Split-mouth designs are a valuable experimental approach wherein a mouth is partitioned into two sides, and these halves are randomly allocated to different treatments. This design offers a notable advantage by minimizing the impact of inter-subject variability on the estimation of treatment effects. The current work employed the Er:YAG laser approach due to its established safety in the photoablation of hard tissue and its excellent water absorption properties, as documented in previous research [17]. 

The histological evaluation conducted by SEM revealed that there were no statistically significant differences in the bond interface quality between restorations placed after conventional caries removal and those placed after Er:YAG laser caries removal in primary molars. A total of 20 teeth from 10 subjects were analyzed in this study, comparing those prepared conventionally with those made using laser technology. The integration of the teeth was assessed at both the enamel–composite interface and the dentin–composite interface within the restoration walls. Several factors can influence the bond interface, including the substrate. Enamel, which mainly consists of inorganic components with a small amount of water, is considered a suitable substrate for bonding to adhesive systems. In contrast, dentin is composed of 47% apatite crystals, 20% water, and 33% organic material. Additionally, regional differences in dentin, such as the inter-tubular area and tubule orientation, can affect the quality of the dentin/adhesive interface [18]. Furthermore, acid etching during dentin conditioning causes a change in the composition of dentin, resulting in an increase in water content from 18% to 50–70% by volume. This alteration has a negative impact on the physical and mechanical properties of the dentin/adhesive interface. Additionally, the etch and rinse technique is linked to variations in the penetration of the adhesive and the action of the conditioning acid agent. This can lead to incomplete hybridization and make dentin collagen more susceptible to hydrolytic degradation and nanoleakage. Furthermore, if the adhesive resin monomer fails to encapsulate the exposed dentinal collagen fibers during the bonding process, the activation of dentinal Matrix Metalloproteinases (MMPs) enzymes might cause the degradation of the dentin/adhesive contact [19]. Furthermore, the constituents of the adhesive system have a direct influence on the quality of the bonding interface. One often utilized component is 2–hydroxyethyl methacrylate (HEMA), which leads to an instant enhancement in the bond strength [20]. However, the durability of the dentin–resin interaction is reduced when HEMA is employed [21,22].

The potential rationale behind the favorable outcomes seen in the current investigation could be attributed to the utilization of an acid etch system featuring 37% phosphoric acid in conjunction with the Clearfil Universal Bond Quick etching primer. According to previous studies, it has been suggested that acid etching of enamel subsequent to laser treatment, serving as a form of enamel conditioning, yielded significantly improved results. After the application of the Er:YAG laser, it is possible that acid etching could lead to the demineralization of the inorganic portion of the dentin surface. This demineralization process may create a conducive environment for the tangling of polymer chains with collagen fibrils [23,24]. The potential benefit of adhesive infiltration may be facilitated by the enlarged pores within the dentinal tubules [25]. An alternative interpretation of these findings posits that the laser beam employed in the experiment was suitably selected and used during clinical procedures. There is a claim that the utilization of an Er:YAG laser with unsuitable parameters leads to the removal of intact dentin, resulting in an uneven dentin surface without a smear layer and exposed dentinal tubules. This, in turn, compromises the effectiveness of adhesive bonding [26].

Four restorations, two conventionally prepared and two laser-prepared, did not achieve integration at the floor of the dentin composite interface. This phenomenon can be ascribed to several factors. First, the impact of the pulpal pressure applied at the cavity floor, the closest point to the pulp, could have played a role. The dentinal tubules located at the base of the cavity exhibit a larger diameter [27]. This enlargement may have resulted in the leakage of dentinal fluids from the openings of the cut dentinal tubules [28,29]. It is important to note that the total etching technique was employed in the present study, which potentially could have removed the smear layer and smear plugs [16]. Consequently, this exposure of the cavity floor interface to the strong pulpal pressure could have led to the contamination of the adhesive system with fluids during and after the polymerization process [28,29]. Second, the use of the total-etch technique in our study may have resulted in the dissolution of a significant portion of the dentin hydroxyapatite, thereby exposing a complex collagen dentinal network that proved challenging for the adhesive resin to infiltrate [16,30]. The formation of a hybrid layer, which lacks resin, has been observed in previous studies. It has been demonstrated that this denuded collagen in the hybrid layer is susceptible to enzymatic attack [13]. Consequently, the hybrid layer gradually disintegrates over time. It is hypothesized that these changes are more pronounced in the cavity floor, which explains why these observations were made early in the cavity floor during the limited clinical follow-up period of the current study. Furthermore, the observed disparity in the bond interface between the walls and the floor of the restorations in our samples can perhaps be attributed to the contrasting bonding durability characteristics of enamel and dentin. The tubules within the dentin are filled with fluid, which is transported through them by means of capillary action. This process is facilitated by the hydrostatic pressure present within the pulp chamber. The wettability of the dentin surface and the existence of intra-pulpal pressure are significant factors in adhesive procedures [31]. The variation in pulpal pressure in vital teeth has been widely recognized, in addition to the distinction in bond strength between pulpal and distant dentin Fields [32]. One additional factor to consider is that certain subjects in the present study necessitated a lesser amount of local anesthesia (LA) administration during dental procedures, which could potentially have a detrimental effect on pulpal pressure. Conversely, the use of LA solutions containing vasoconstrictors has been found to decrease local blood pressure, thereby potentially reducing pulpal pressure [33]. In physiological conditions, it has been observed that the average value of this pressure ranges from approximately 30 to 40 cm H_2_O. The removal of the smear layer has been shown to cause an augmented outward flow of tubular fluid. This phenomenon is believed to occur due to the pressure exerted, which aims to prevent resin monomers from penetrating dentin surfaces. The extent of this effect may vary depending on the specific monomers employed [32]. The two participants who showed compromised bond interface at the pulpal floor (four teeth) included in our investigation did not have local anesthesia (LA) during either the Er:YAG laser or conventional intervention. This lack of anesthesia administration may account for the observed failure to achieve integration of the dentin–composite interface at the base of the restorations in these four samples.

The results of our research align with many prior investigations. In their study, Wright et al. (1993) conducted a comparison of microleakage in cavity preparations that were traditionally created with a high-speed drill and acid-etched vs. those that were etched after being prepared with an Er:YAG laser. Through the utilization of SEM evaluation, researchers discovered that the process of preparation and etching using an Er:YAG laser does not have a detrimental impact on the microleakage in the vicinity of the dental restoration [34]. Visuri et al. (1996) conducted a comparison of the bonding properties of composite materials and dentin after preparing the dentinal surface using two different methods: an Er:YAG laser; and a normal dental bur. Additionally, the researchers investigated the effects of an acid-etching treatment on the bonding process. Scanning electron microscopy (SEM) images revealed the presence of exposed tubules on the surfaces subsequent to the laser treatment. Additionally, it was shown that acid etching also led to the exposure of tubules, reaching the determination that the utilization of Er:YAG laser for dentin preparation yielded a surface that was conducive to the effective bonding of composite material [35]. Yamada et al. (2002) examined the surface morphology of cavities created using Er:YAG laser irradiation. They then conducted a comparative analysis of the extent of microleakage following composite resin repair between these laser-prepared cavities and bur-prepared cavities in primary teeth. The investigation was carried out in an in vitro setting. The results of their study indicated that there were no statistically significant disparities observed in the microleakage of composite resin restorations between laser-prepared cavities and bur-prepared cavities. The cross-sectional analysis of the cavities without any microleakage revealed a satisfactory bond between the restorative material and the dental hard tissues. Furthermore, it is worth noting that there was a lack of gap seen at the interface. The researchers reached the determination that cavities created using the Er:YAG laser exhibited the ability to reduce microleakage in composite resin restorations within the primary teeth [36]. In 2010, Ramos et al. conducted a study to examine the impact of Er:YAG laser irradiation on dentinal collagen using transmission electron microscopy. Additionally, they evaluated the resin–dentin contact by scanning electron microscopy and reached a conclusion that the application of Er:YAG laser in the ablation of human dentin did not result in any significant changes to the primary adhesion parameters in comparison to those achieved using traditional methods [37]. In 2013, Oznurhan et al. conducted an in vitro analysis of the hybrid layer and nanoleakage observed in composite resin restorations placed on primary teeth. These restorations were placed in cavities that were produced using either an Er,Cr:YSGG laser or a bur, followed by acid etching. The most favorable outcomes were observed when laser preparation was employed in conjunction with acid etching [38].

In contrast, the findings of our study are inconsistent with those of several previous investigations. De Munck et al. conducted an assessment to test the notion that tooth substrate, prepared using either an Er:YAG laser or a diamond bur, has equivalent receptiveness to adhesive techniques. The composite was bonded using two different adhesives: OptiBond FL, a total-etch adhesive, applied with and without preceding acid-etching; and Clearfil SE Bond, a self-etch adhesive. The analysis of failure patterns was conducted utilizing a stereomicroscope, and subsequent processing of samples was performed to facilitate examination using a Field Emission Scanning Electron Microscope (Fe–SEM). The authors reached the conclusion that cavities made using laser technology exhibited reduced receptiveness to adhesive processes compared to cavities prepared using conventional bur-cut methods [39]. In 2004, Ramos et al. conducted an in vitro study to examine the impact of Er:YAG laser on dentin bonding and the interaction patterns of various adhesive systems with the laser-treated substrate. That study aimed to assess the tensile bond strength of three different adhesive systems, namely, Clearfil SE Bond (CSEB), Single Bond, and Gluma One Bond (GOB), on both lased and non-lased dentin surfaces. Additionally, the adhesive interface morphology was studied using scanning electron microscopy (SEM). The researchers discovered that the application of Er:YAG laser irradiation had a significant negative impact on the development of uniform resin–dentin hybridization zones and resulted in decreased bond strengths. The CSEB self-etching primer displayed the greatest susceptibility to laser ablation on the dentin substrate, leading to the least favorable adhesion outcome [40]. The study conducted by Bakry et al. (2009) aimed to assess the impact of Er:YAG laser ablation on the nanoleakage of Er:YAG-lased dentin that was bonded to a self-etching adhesive system, both with and without the application of pulpal pressure, using an in vitro experimental approach. The researchers noticed distinct variations in the nanoleakage patterns at the dentin/bond contact, depending on whether the dentin underwent traditional preparation or was ablated using an Er:YAG laser. It was determined that the application of Er:YAG laser ablation on dentin had a negative impact on the sealing efficacy of SE Bond when bonded to dentin under simulated pulpal conditions [10]. The study conducted by Moretto et al. (2011) aimed to assess the impact of erbium laser irradiation on the morphology of dentin using an in vitro experimental approach. The scanning electron microscopy (SEM) images exhibited a discernible arrangement of altered markers accompanied by circular formations surrounding them. It was observed that the application of erbium laser irradiation on dental hard tissue led to the development of a distinct morphological pattern in dentin and collagen fibrils. This pattern has been proven to have a detrimental impact on the bond strength between the dental hard tissue and composite resin [41]. The study conducted by Ozel et al. (2016) aimed to examine the impact of two different cavity-filling techniques on the occurrence of microleakage in class II resin restorations that were made using both Er:YAG laser and diamond bur. It was shown that the cavities created with an Er:YAG laser exhibited higher levels of microleakage compared to traditionally prepared cavities employing burs. This finding was consistent across different restorative materials and was observed at both the occlusal and cervical margins [42].

The clinical significance of the present study lies in the fact that the clinical success of the restorations used in this study was investigated and reported in a previous study, using precise clinical and radiographic evaluation standards, which resulted in outstandingly successful restorations [5]. The current research revealed instances of bond interface failures in both conventional and Er:YAG laser-prepared cavity preparations, underscoring the challenge of precisely assessing the bonding interface, which was a part of the success of the restoration, particularly the dentin floor interface, in a clinical setting using clinical assessment tools. In other words, the restoration may seem to be successful from a clinical perspective, but some failures in the bond interface may go unnoticed unless observed using a scanning electron microscope (SEM). This underscores the necessity of considering alternative precise clinical instruments, such as Optical Coherence Tomography (OCT), for the correct identification and assessment of dental restoration success.

This study has a number of limitations, like any other research. The accuracy of estimating the exfoliation time of primary teeth (dental age) was found to be inconsistent in all cases when compared to the chronological age of the participants at the one-year follow-up. For this reason, only 20 of the 70 teeth that were initially present could be used for the analysis in the follow-up study after a year. Furthermore, the number of teeth included in the final analysis was unexpectedly small, which might have compromised the statistical power and raised the possibility of a Type II error. The diameters of the cavities on the paired primary molars within the same subject exhibited heterogeneity, potentially contributing to differences observed between groups. Conversely, the research exhibited various methodological advantages. The utilization of a split-mouth design trial in clinical investigations was found to be a highly effective approach. This design lets each participant serve as their own control, hence minimizing inter-subject variability in the estimation of treatment effects. Consequently, this method yielded reliable and valid data. Furthermore, the incorporation of in vivo and in vitro experimental methodologies represents an additional advantage of our study. The inclusion of a one-year period during which the patients’ teeth are in functional use prior to examination under scanning electron microscopy (SEM) constitutes a great contribution to enhancing the accuracy of our findings.

Additional research is required to investigate the precise impact of pulpal pressure on the floor of cavities, particularly studies with extended follow-up periods. In contemporary clinical practice, the availability of instruments capable of promptly identifying indications of composite restoration failure, particularly within the interface region, holds significant significance. Additional in vivo investigations are warranted to assess the longevity and efficacy of laser restorations in various cavity preparations over an extended duration. Further investigations involving in vitro and in vivo research are warranted to assess the bond interface quality of laser restorations in a more extensive sample size and over an extended duration in order to provide additional support for the findings presented in this study. In order to substantiate the existing findings, it is recommended that further in vitro and in vivo studies be undertaken to examine the effects of local anesthesia (LA) administration on pulpal pressure and its implications on the quality of the bond interface in laser restorations. These investigations should encompass a larger sample size and an extended length of observation. Additional in vitro and in vivo investigations are warranted to evaluate the influence of total-etch and self-etch systems subsequent to the Er:YAG treatment on the bond interface quality of laser restorations. These studies should encompass a larger sample size and an extended duration to validate the findings reported in this study.

## 5. Conclusions

After one year, the bond interface quality according to SEM evaluation of class I restorations placed in primary molars prepared using the Er:YAG laser performed as well as cavities prepared using conventional rotary burs.

## Figures and Tables

**Table 1:** Within-subject comparison of dentin floor bond interface quality of restorations placed after the conventional and Er:YAG laser methods.

Integration of the Dentin Floor with the Adhesive		Conventional Caries Removal		*p*-Value
		Integrated	Not Integrated	
Laser caries removal	Integrated	6 (60.0%)	2 (20.0%)	0.617
	Not integrated	2 (20.0%)	0	

McNemar test.

## Data Availability

The data of this study can be obtained upon request to the corresponding author at omfelemban@kau.edu.sa.
